# Musculoskeletal Benefits from a Physical Activity Program in Primary School are Retained 4 Years after the Program is Terminated

**DOI:** 10.1007/s00223-021-00853-0

**Published:** 2021-04-29

**Authors:** Björn E. Rosengren, Erik Lindgren, Lars Jehpsson, Magnus Dencker, Magnus K. Karlsson

**Affiliations:** 1 Clinical and Molecular Osteoporosis Research Unit, Department of Orthopedics, Skane University Hospital (SUS), 20502 Malmo, Sweden; 2 Department of Physiology, Clinical Sciences, Lund University, Skane University Hospital, Malmo, Sweden

**Keywords:** Bone mineral content, Bone mineral density, Children, Muscle strength, Physical activity

## Abstract

Daily school physical activity (PA) improves musculoskeletal traits. This study evaluates whether the benefits remain 4 years after the intervention. We followed 45 boys and 36 girls who had had 40 min PA/school day during the nine compulsory school years and 21 boys and 22 girls who had had 60 min PA/school week (reference), with measurements at baseline and 4 years after the program terminated. Bone mineral content (BMC; g) and bone mineral density (BMD; g/cm^2^) were measured by dual-energy X-ray absorptiometry and knee flexion peak torque relative to total body weight (PT_flex_TBW) at a speed of 180 degrees/second with a computerized dynamometer. Group differences are presented as mean differences (adjusted for sex and duration of follow-up period) with 95% confidence intervals. The total gain bone mass [mean difference in spine BMC +32.0 g (14.6, 49.4) and in arms BMD of +0.06 g/cm^2^ (0.02, 0.09)] and gain in muscle strength [mean difference in PT_flex180_TBW +12.1 (2.0, 22.2)] were greater in the intervention than in the control group. There are still 4 years after the intervention indications of benefits in both bone mass and muscle strength gain. Daily school PA may counteract low bone mass and inferior muscle strength in adult life. **ClinicalTrials.gov.**NCT000633828 retrospectively registered 2008-11-03

## Introduction

Thirty percent of children suffer a fracture before the age of 18 [1] and 50% of women and 22% of men after the age of 50 [2]. This results in enormous costs for society [3]. Several risk factors for fracture have been identified [4, 5], some of which are modifiable and possible to target by intervention. Regular physical activity (PA) is one such factor [6–10]. PA intervention is easy to implement at low cost, accessible, without adverse side effects, and possible to implement on population level. A high level of PA during childhood and adolescence has also been associated with beneficial development in bone mass, neuromuscular function, muscle strength, and a gradually lower fracture incidence [6–11], as well as low fracture incidence in adulthood [12–16]. These benefits are also apparent after moderately intense PA intervention, on a level that enables all children to participate [6–10]. PA interventions are probably best initiated early in life, as the greatest skeletal response to mechanical load occurs during the pre- and early pubertal years [17], and as 25% of the adult bone mass is acquired during two pubertal years [18]. Finally, if PA interventions during childhood and adolescence lead to higher peak bone mass (PBM), the clinical significance would be even greater, as 50% of the variance in bone mineral density (BMD) in old age seems to be predicted by peak bone mass [19] and a 10% increase in PBM may delay osteoporosis by 13 years [20]. Thus, a high level of PA during childhood and adolescence, at least hypothetically, may be a way to counteract low bone mass and high fracture incidence in adulthood.

Some studies infer that a cessation of high level of PA is followed by greater loss in bone mass than expected by age [16, 21, 22]. Others, however, found that bone mass benefits are retained [12–16, 23, 24]. Due to the conflicting results, there is a need for longitudinal controlled studies that follow individuals from before PA intervention, during the period with PA intervention and after the termination of PA intervention.

We hypothesized that PA-induced musculoskeletal benefits gained during childhood and adolescence remain in young adulthood but are attenuated after termination of the program. The primary research question was whether the musculoskeletal benefits previously found in the POP cohort at the end of the intervention [6] are attenuated after termination of the program. Secondarily we asked if the intervention, with inclusion of the 4-year post-intervention period, still was associated with beneficial gain in musculoskeletal traits.

## Materials and Methods

The Pediatric Osteoporosis Prevention (POP) study is a population-based, prospective, controlled exercise intervention study with the primary aim of investigating whether daily school-based PA improves musculoskeletal development traits and reduces fracture risk. The project has been described in detail previously [6–10]. Briefly, the POP cohort includes children from four government-funded, community-based elementary schools in the same geographical area with a uniform socioeconomic status. Before study start, all schools had the same amount of physical education (60 min/school week). The first school that accepted participation was assigned as the intervention school and the remaining three schools served as control schools. In the intervention school, the amount of PA was increased to 200 min/school week (daily school classes of 40 min during all nine compulsory school years). The PA included moderate activities, all used in the regular physical education (PE) curriculum such as gymnastics, team sports, dancing, running, jumping and free PA activities. In Sweden, PE is a compulsory school subject and all children thus participated in the increased PE. The control schools used the same activities but continued with national standard of 60 min/school week (provided in 1–2 classes/week) during the same period. The regular schoolteachers continued to lead the PE classes. We did not register the proportion of different activities, the proportion of impact and endurance activities, the individual participation rate, or participation intensity.

**Fig. 1:**
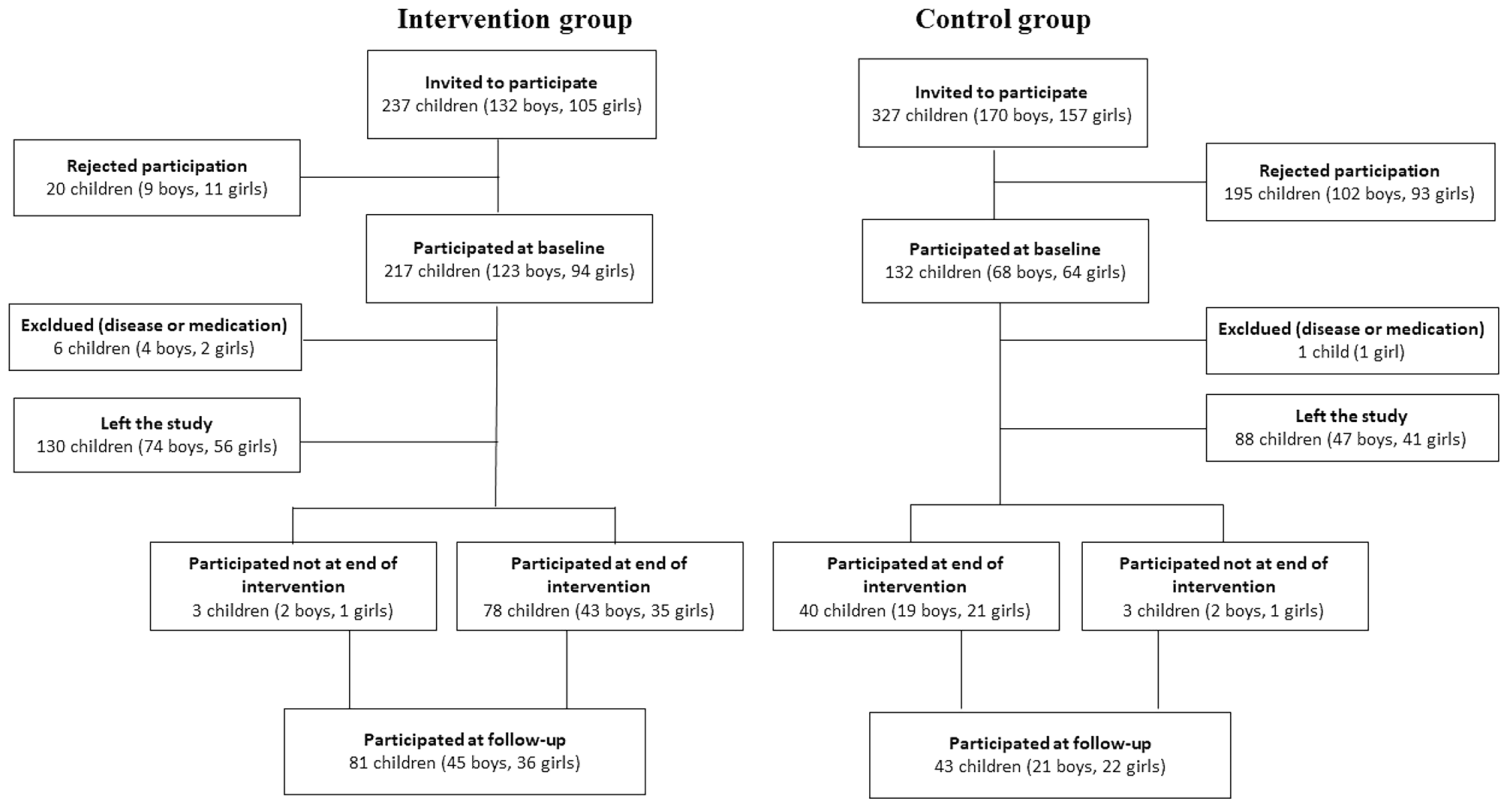
Flowchart of study participants

At the start of the POP study, we invited all children in the four above-mentioned schools who began first grade in 1998–2000 to the study. The children were then 6–9 years old, and 98% were of Caucasian ethnicity [25]. 217/237 children (123 boys and 94 girls) in the intervention school and 132/327 (68 boys and 64 girls) in the control schools accepted participation. We excluded four boys and two girls in the intervention and one girl in the control schools who had chronic disease or medication that could interfere with bone growth. This rendered 342 children included at baseline. Previous reports have shown that the children with daily school PA during the intervention had higher gains in bone mass and muscle strength than children without intervention [7–10], also through puberty [6]. In the current study we re-evaluated the children a mean 4 years (range 3–5) after termination of the intervention. Thirty-seven individuals, 22 in the intervention group, and 15 in the control group, moved during the study period, leaving 305 participants still alive and living in the region. A total of 124 individuals (36% of all those who participated at baseline 11 years previously and 41% of those still living in the region) attended the 4-year post-intervention exam (Fig. 1).

The re-evaluation included the same measurements as the examinations at baseline and at the end of the intervention [6–10]. Bone mineral content (BMC; g) and bone mineral density (BMD; g/cm^2^) in total body less head, arms, legs, spine and left femoral neck (FN) were measured by dual-energy X-ray absorptiometry (DXA). We used DPX-L® version 1.3z, Lunar Corporation, Madison, WI, USA at baseline and at end of intervention and DXA-iDXA® version enCore 13.60, Lunar Corporation, Madison, WI, USA in 55 individuals (33 from intervention group and 22 from control group) and DXA-Prodigy® version enCore 9.30, Lunar Corporation, Madison, WI, USA in 69 individuals (48 from intervention group and 21 from control group) at the 4-year postintervention exam. The DXA apparatus was calibrated daily during the entire study period by use of a phantom and we found no long-term drift in the equipment. We measured muscle strength as concentric isokinetic peak torque (PT) (Nm) by a computerized dynamometer (Biodex System III Pro®, Biodex Medical Systems Inc. Shirley, NY, USA) and from this calculated isokinetic PT related to total body weight (PTTBW) as [(PT/body weight) × 100] for right knee flexion (_flex_) at speeds of 60 and 180 degrees/second. We used the highest PT value of five repeated movements of flexion. Research technicians performed all measurements. The coefficients of variation (%), evaluated by duplicate measurements in 13 healthy children, were 1.4–5.2% for BMC, 2.4–2.6% for aBMD, 6.7% for PT_flex60_ and 9.1% for PT_flex180_.

Body height (cm) was measured with a Holtain Stadiometer (Holtain LTD, Pembrokeshire, UK) and body mass (kg) with an electric scale (Avery Berkel HL 120 Electric Scale, Avery Berkel, West Midlands, UK). Body mass index (BMI) was calculated as body weight divided by body height squared (kg/m^2^). A research nurse assessed Tanner stage [25] at baseline, while we used self-assessment at follow-up. All children were in Tanner stage I at baseline and in Tanner stage V at follow-up. Lifestyle (dairy intake, alcohol use, smoking), medical conditions (asthma, achondroplasia, epilepsy, kidney disease, thyroid disease, diabetes, bowel disease), medication (cortisone, levaxine, liothyronine, insulin, antiepileptic drugs, antidepressants and oral contraception pills), school PA and duration of weekly organized leisure-time PA were evaluated at the examinations through a non-validated questionnaire, at baseline with the help of the parents [6–10].

We undertook two drop-out analyses. In the first we used data collected at the compulsory Swedish first-grade school health examinations. We compared height, weight and BMI in children who accepted participation in the study with those who declined. We found no clinically relevant (or statistically significant) differences between the groups [10]. In the second drop-out analyses we compared anthropometry, BMC, BMD and PT in the participants who attended baseline and the 4-year postintervention exam with the participants who attended only the baseline exam (but not the 4-year postintervention exam). These analyses likewise revealed no clinically relevant (or statistically significant) differences between the groups (Table 1).

**Table 1:** Second drop-out analysis with comparison of baseline values (at school start) between participants who attended the baseline as well as the 4-year postintervention exam and participants who attended only the baseline (but not the 4-year postintervention exam). Anthropometry was measured by standard equipment

	Boys (*n* = 187)			Girls (*n* = 155)		
	Participants (*n* = 66)	Drop-outs (*n* = 121)	*p*-value	Participants (*n* = 58)	Drop-outs (*n* = 97)	*p*-value
At school start (baseline)						
Age	7.7 ± 0.6	7.7 ± 0.6	0.55	7.6 ± 0.6	7.7 ± 0.6	0.74
Anthropometry						
Height (cm)	129.0 ± 7.2	128.7 ± 6.2	0.78	127.2 ± 6.9	128.5 ± 7.0	0.25
Weight (kg)	27.6 ± 5.1	27.8 ± 5.9	0.84	26.7 ± 5.5	27.6 ± 5.2	0.31
BMI (kg/m^2^)	16.5 ± 2.2	16.6 ± 2.4	0.70	16.4 ± 2.3	16.7 ± 2.5	0.47
Bone mineral content (BMC; g)						
Total body less head	655.0 ± 154.8	654.2 ± 153.4	0.97	607.4 ± 139.9	626.3 ± 136.7	0.42
Arms	88.7 ± 20.5	87.2 ± 19.7	0.62	79.1 ± 17.7	81.3 ± 17.4	0.47
Legs	282.3 ± 72.7	284.1 ± 72.2	0.87	268.3 ± 64.5	280.4 ± 67.0	0.28
Spine	85.1 ± 20.3	84.9 ± 20.8	0.95	80.0 ± 18.7	81.0 ± 17.6	0.74
Hip – femoral neck	2.8 ± 0.6	2.9 ± 0.7	0.38	2.6 ± 0.7	2.6 ± 0.5	0.76
Bone mineral density (BMD; g/cm^2^)						
Total body less head	0.69 ± 0.06	0.69 ± 0.06	0.71	0.68 ± 0.05	0.69 ± 0.05	0.82
Arms	0.62 ± 0.05	0.61 ± 0.04	0.47	0.60 ± 0.05	0.60 ± 0.04	0.55
Legs	0.76 ± 0.08	0.75 ± 0.07	0.69	0.75 ± 0.07	0.75 ± 0.07	0.68
Spine	0.69 ± 0.06	0.68 ± 0.06	0.50	0.69 ± 0.07	0.68 ± 0.06	0.78
Hip – femoral neck	0.77 ± 0.12	0.78 ± 0.10	0.57	0.71 ± 0.11	0.72 ± 0.08	0.61
Peak torque muscle strength (Nm)						
Knee flexion (60°)	23.0 ± 6.6	23.2 ± 7.3	0.85	21.9 ± 5.6	21.8 ± 5.6	0.93
Knee flexion (180°)	21.0 ± 6.0	21.1 ± 6.2	0.85	19.6 ± 5.6	19.6 ± 5.1	0.96

Bone mineral content (BMC), bone mineral density (BMD), bone size, and soft tissue composition were measured with Dual Energy X-Ray Absorptiometry (DXA). Muscle strength was measured by Biodex®. Data are presented as absolute numbers (n), means ± standard deviations

We used Statistica® version 12.0 (Statsoft Inc®) for statistical analyses and present descriptive data as absolute numbers (n), proportions (%), means with standard deviations (SD), and inferential statistics as age-adjusted mean differences with 95% confidence intervals (95% CI). We calculated study period changes as (i) follow-up values minus the values at the end of the intervention and (ii) follow-up value minus the baseline values. Analysis of covariance (ANCOVA), adjusted for the proportion of boys and girls and duration of follow-up period, was used to compare group differences in trait changes in the two periods. Interaction term was also included (sex and group) to evaluate whether the intervention conferred different effects in boys and girls. We used mean ± SD derived from the control cohort to express differences between groups. We regarded *p* < 0.05 as a statistically significant difference. All children in the POP study and/or their parents/guardians provided written consent before participation. The study was approved by the Ethics Committee of Lund University, Sweden (LU 453-98; 1998-09-15) and is registered as a clinical trial (ClinicalTrials.gov.NCT000633828).

## Results

### Sex-Specific Group Characteristics at Baseline

Baseline sex-specific group characteristics are presented in Table 2.

**Table 2:** Lifestyle characteristics, anthropometry, bone mass, and muscle strength in the intervention (*n* = 81) and control (*n* = 43) cohorts at study start (school start)

	Boys (*n* = 66)			Girls (*n *= 58)		
	Intervention (*n* = 45)	Control (*n* = 21)	Mean difference (age-adjusted)	Intervention (*n* = 36)	Control (*n* = 22)	Mean difference (age-adjusted)
Lifestyle						
Age (years)	7.5 ± 0.6	8.0 ± 0.7	n.a	7.5 ± 0.6	7.9 ± 0.6	n.a
Exclusion of dairy products	0/45 (0%)	3/21 (14%)	n.a	0/35 (0%)	1/21 (5%)	n.a
Chronic medical conditions	7/45 (16%)	0/21 (0%)	n.a	2/35 (6%)	1/19 (5%)	n.a
Current medication	1/45 (2%)	0/21 (0%)	n.a	1/35 (3%)	2/22 (9%)	n.a
Total organized PA	6.4 ± 3.5	4.3 ± 3.4	2.1 (0.1, 4.1)	4.9 ± 1.7	3.2 ± 1.7	2.1 (1.1, 3.1)
Anthropometry						
Height (cm)	128.6 ± 7.3	130.0 ± 7.0	1.7 (− 1.5, 5.1)	126.6 ± 6.4	128.2 ± 7.6	1.5 (− 1.5, 4.5)
Weight (kg)	27.8 ± 5.5	27.4 ± 4.5	2.3 (− 0.2, 4.8)	26.5 ± 5.3	27.0 ± 6.1	0.8 (− 2.3, 3.8)
BMI (kg/m^2^)	16.7 ± 2.5	16.1 ± 1.5	1.0 (− 0.2, 2.2)	16.5 ± 2.5	16.2 ± 2.1	0.2 (− 1.1, 1.6)
Bone mineral content (BMC; g)						
Total body less head	651.0 ± 164.0	663.4 ± 136.3	52.2 (− 21.0, 125.4)	599.5 ± 133.0	620.5 ± 153.2	28.4 (− 44.7, 101.6)
Arms	88.9 ± 21.5	88.4 ± 18.7	8.1 (− 2.0, 18.2)	77.9 ± 17.2	81.1 ± 18.7	2.2 (− 7.3, 11.8)
Legs	280.0 ± 75.7	287.3 ± 67.5	24.4 (− 9.2, 58.0)	261.6 ± 59.9	279.4 ± 71.6	8.3 (− 23.4, 40.1)
Spine	85.3 ± 21.9	84.5 ± 16.7	8.4 (− 1.5, 18.3)	81.3 ± 18.9	77.7 ± 18.5	9.3 (− 0.8, 19.4)
Hip – femoral neck	2.8 ± 0.7	2.8 ± 0.5	0.2 (− 0.1, 0.5)	2.5 ± 0.7	2.7 ± 0.8	0.0 (− 0.4, 0.4)
Bone mineral density (BMD; g/cm^2^)						
Total body less head	0.69 ± 0.06	0.69 ± 0.06	0.02 (− 0.01, 0.05)	0.70 ± 0.05	0.69 ± 0.05	0.00 (− 0.03, 0.03)
Arms	0.62 ± 0.05	0.61 ± 0.05	0.02 (− 0.00, 0.05)	0.60 ± 0.05	0.60 ± 0.05	0.01 (− 0.02, 0.04)
Legs	0.76 ± 0.08	0.76 ± 0.08	0.03 (− 0.01, 0.07)	0.74 ± 0.06	0.77 ± 0.07	− 0.01 (− 0.05, 0.02)
Spine	0.69 ± 0.07	0.70 ± 0.05	0.01 (− 0–02, 0.04)	0.69 ± 0.06	0.69 ± 0.08	0.00 (− 0.04, 0.04)
Hip – femoral neck	0.77 ± 0.12	0.78 ± 0.13	0.04 (− 0.03, 0.10)	0.71 ± 0.11	0.72 ± 0.10	0.02 (− 0.04, 0.08)
Muscle strength – peak torque (Nm)						
Knee flexion 60°	22.2 ± 7.0	24.8 ± 5.1	− 0.1 (− 3.2, 3.0)	20.5 ± 5.5	24.0 ± 5.4	− 2.1 (− 5.0, 0.9)
Knee flexion 180°	20.0 ± 6.1	23.1 ± 5.4	− 1.1 (− 4.1, 1.8)	18.7 ± 5.7	21.2 ± 5.1	− 1.6 (− 4.8, 1.5)
Muscle strength – peak torque TBW (Nm/kg)*100						
Knee flexion 60°	80.5 ± 19.0	92.5 ± 15.5	− 8.4 (− 18.3, 1.5)	78.8 ± 15.8	90.0 ± 21.1	− 9.5 (− 17.8, 1.2)
Knee flexion 180°	73.1 ± 17.7	84.9 ± 7.2	− 9.5 (− 18.2, − 0.8)	72.7 ± 21.4	80.3 ± 16.5	− 8.0 (− 19.4, 3.49

Data are presented as numbers (n), proportions (%), means ± standard deviations, or mean age-adjusted difference with 95% confidence intervals

### Changes in Musculoskeletal Traits During the 4 Years Following Termination of the Intervention

**Fig. 2:**
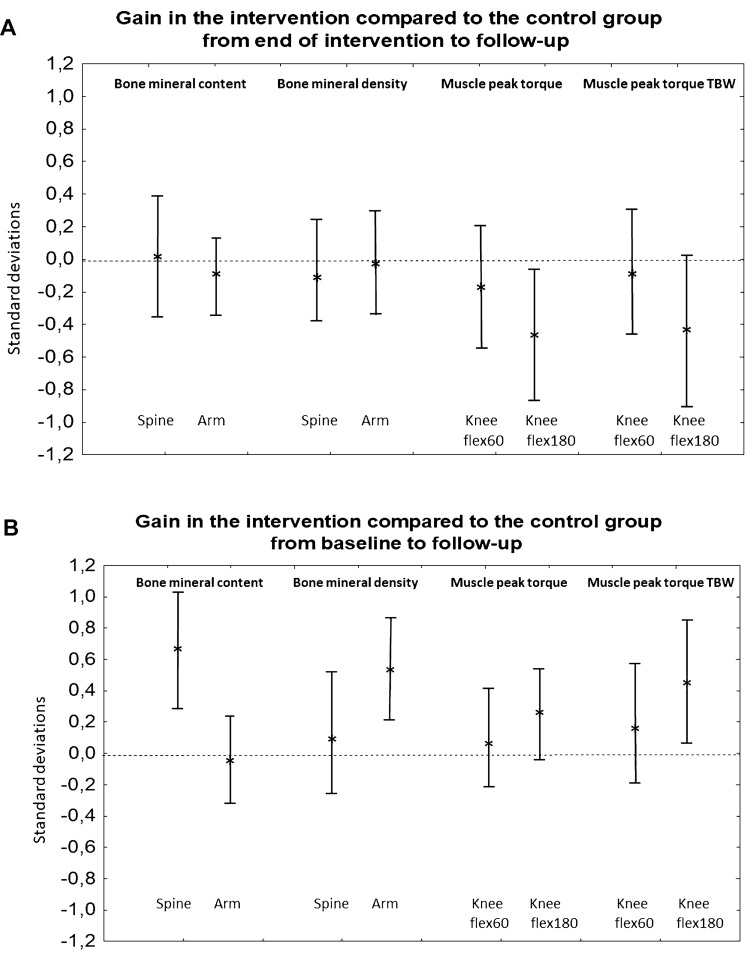
Gains in musculoskeletal traits in the intervention group compared to the control group from **a** end of the intervention to follow-up and **b** from baseline to follow-up. Data are shown for bone mineral content (BMC) and bone mineral density (BMD) in total body less head and femoral neck and muscle strength as knee flexion peak torque 180 degrees/second and knee flexion peak torque 180 degrees/second relative to total body weight (TBW). Bars represent the mean group deviation (adjusted for sex and duration of follow-up period) in the intervention group compared to mean gain in the control group (0.0) expressed in standard deviations (SD) with 95% confidence intervals

We found similar developments in BMC or BMD in former intervention and former control children during the 4 years that followed termination of the intervention (all *p* > 0.05) (Table 3). In contrast, after termination of the intervention, muscle strength development was inferior in children in the former intervention group to that of children in the control group [mean difference in PT_flex180_ changes –5.6 Nm (–10.5, –0.8)] (Table 3). This corresponds to an attenuation of –0.5 (–0.8, –0.1) standard deviations (SD) (Fig. 2a). Interaction terms pointed to similar effects in boys and girls during the 4-year period that followed termination of the intervention (all *p* > 0.05).

**Table 3:** Changes in musculoskeletal trait from end of the intervention period to mean 4 (range 3–5) years after termination of the intervention and from the baseline to the follow-up exam, a period of mean 11 (range 10–12) years

	Changes from end of intervention to mean 4 years (range 3–5) after termination of the intervention (*n* = 118)				Changes from baseline to study end (*n* = 124)			
	Intervention (*n* = 78)	Control (*n* = 40)	Mean difference (adjusted)	p-value (adjusted)	Intervention (*n* = 81)	Control (*n* = 43)	Mean difference (adjusted)	p-value (adjusted)
Bone mineral content (BMC; g)								
Total body less head	189.1 (136.2, 242.0))	199.0 (124.2, 273.8)	− 9.9 (− 104.2, 84.5)	0.84	1727.3 (1654.2, 1800.4)	1615.5 (1512.1, 1919.0)	111.8 (− 20.4, 244.0)	0.10
Arms	86.6 (78.5, 94.6)	91.1 (79.6, 102.5)	− 4.5 (− 18.9, 9.9)	0.54	285.7 (274.9, 296.7)	286.6 (271.2, 302.0)	− 0.9 (− 20.6, 18.9)	0.93
Legs	117.8 (97.9, 137.6)	126.3 (98.2, 154.4)	− 8.5 (− 44.0, 26.9)	0.63	800.9 (769.0, 832.7)	771.6 (726.5, 816.7)	29.2 (− 28.4, 86.9)	0.32
Spine	− 33.6 (− 45.4, − 21.9)	− 33.7 (− 50.3, − 17.1)	0.1 (− 20.8, 21.0)	0.99	**160.6 (151.0, 170.1)**	**128.6 (114.9, 142.2)**	**32.0 (14.6, 49.4)**	** < 0.001**
Hip – femoral neck	0.3 (0.1, 0.4)	0.2 (0.0, 0.4)	+0.0 (− 0.2, 0.3)	0.76	3.1 (2.9, 3.2)	2.9 (2.6, 3.1)	0.2 (− 0.1, 0.5)	0.24
Bone mineral density (BMD; g/cm^2^)								
Total body less head	0.05 (0.03, 0.06)	0.05 (0.03, 0.07)	0.00 (− 0.02, 0.02)	0.76	0.39 (0.38, 0.41)	0.38 (0.36, 0.40)	0.02 (− 0.01, 0.05)	0.27
Arms	0.00 (− 0.02, 0.02)	0.01 (− 0.02, 0.03)	0.00 (− 0.04, 0.04)	0.93	**0.25 (0.24, 0.27)**	**0.20 (0.18, 0.22)**	**0.06 (0.02, 0.09)**	** < 0.001**
Legs	0.10 (0.09, 0.12)	0.10 (0.08, 0.13)	0.00 (− 0.03, 0.03)	0.98	0.56 (0.54, 0.59)	0.53 (0.50, 0.57)	0.03 (− 0.01, 0.07)	0.17
Spine	0.05 (0.03, 0.06)	0.05 (0.03, 0.07)	− 0.01 (− 0.03, 0.02)	0.68	0.38 (0.37, 0.40)	0.37 (0.34, 0.40)	0.01 (− 0.02, 0.05)	0.42
Hip – femoral neck	0.07 (0.05, 0.09)	0.07 (0.04, 0.10)	+0.00 (− 0.03, 0.04)	0.94	0.37 (0.35, 0.40)	0.37 (0.33, 0.41)	0.00 (− 0.05, 0.05)	0.91
Muscle strength – peak torque (Nm)								
Knee flexion 60°	12.9 (9.4, 16.4)	15.6 (10.7, 20.5)	− 2.7 (− 9.0, 3.5)	0.38	70.3 (65.9, 74.6)	67.8 (61.8, 73.9)	2.4 (− 5.3, 10.1)	0.54
Knee flexion 180°	**4.7 (2.0, 7.5)**	**10.4 (6.5, 14.2)**	**− 5.6 (− 10.5, − 0.8)**	**0.02**	48.6 (45.1, 52.0)	42.6 (37.8, 47.4)	6.0 (− 0.1, 12.1)	0.05
Muscle strength – peak torque TBW (Nm/kg)*100								
Knee flexion 60°	0.7 (− 5.2, 6.7)	2.3 (− 6.0, 10.6)	− 1.6 (− 12.1, 9.0)	0.77	49.5 (43.4, 55.6)	43.4 (34.8, 52.0)	6.1 (− 4.8, 17.1)	0.27
Knee flexion (180°)	− 8.6 (− 13.9, − 3.2)	0.1 (− 7.4, 7.5)	− 8.6 (− 18.1, 0.9)	0.07	**23.2 (17,5, 28.8)**	**11.0 (3.1, 19.0)**	**12.1 (2.0, 22.2)**	**0.02**

Three children in the intervention group and three in the control group missed the evaluation at the end of the intervention period. Data are presented as mean differences (95% confidence intervals) adjusted for proportion of boys and girls and duration of follow-up period in an ANCOVA analyses. Statistically significant group differences are bolded

### Development in Musculoskeletal Traits During the Entire 11-Year Study Period

Even though the muscle strength development was attenuated after the termination of the intervention, we still found a greater gain in muscle strength in the intervention than in the control children during the entire 11-year study period [mean difference in PT_flex180_TBW changes +12.1 (2.0, 22.2)] (Table 3). This corresponds to a 0.5 (0.1, 0.9) SD higher muscle gain (Fig. 2b). Furthermore, the beneficial gain in bone mass that had occurred from study start to the end of the intervention period [6] also remained 4 years after the program terminated, for both BMC [mean difference in spine changes +32.0 g (14.6, 49.4)] and BMD [mean difference in arms changes +0.06 g/cm^2^ (0.02, 0.09)] (Table 3). This corresponds to 0.7 (0.3, 1.1) SD higher gain in BMC and 0.6 (0.3, 0.9) SD higher gain in BMD (Fig. 2b). Interaction terms pointed to similar effects in boys and girls during the entire study period (all *p* > 0.05).

### Sex-Specific Group Characteristics at Age 19

Sex-specific group characteristics at study end are presented in Table 4. A mean 4 years after termination of the intervention, children with former daily school PA still had a higher duration of total PA than control children, with an age- and sex-adjusted difference of +1.9 (+0.0, 3.8) hours/week.

**Table 4:** Lifestyle characteristics, anthropometry, bone mass, and muscle strength in the intervention (*n* = 81) and control (*n* = 43) cohorts at study end, mean 4 (range 3–5) years after termination of the intervention

	Boys (*n* = 66)			Girls (*n* = 58)		
	Intervention (*n* = 45)	Control (*n* = 21)	Mean difference (age-adjusted)	Intervention (*n* = 36)	Control (*n* = 22)	Mean difference (age-adjusted)
Lifestyle						
Age (years)	18.8 ± 0.2	18.8 ± 0.3	n.a	18.7 ± 0.4	18.7 ± 0.3	n.a
Exclusion of dairy products	1/45 (2%)	1/21 (5%)	n.a	3/35 (9%)	0/22 (0%)	n.a
Chronic medical conditions	11/45 (24%)	0/21 (0%)	n.a	6/35 (17%)	0/22 (0%)	n.a
Hormonal birth controls	n.a	n.a	n.a	17/35 (49%)	15/22 (68%)	n.a
Current medication	1/45 (2%)	0/21 (0%)	n.a	2/35 (6%)	1/22 (5%)	n.a
Smoker	3/45 (7%)	5/21 (24%)	n.a	7/35 (20%)	5/22 (23%)	n.a
Teetotaler	1/45 (2%)	1/21 (5%)	n.a	5/35 (14%)	4/22 (18%)	n.a
Total organized PA (hours/week)	7.5 ± 6.5	4.4 ± 2.2	3.2 (0.0, 6.4)	4.3 ± 2.6	3.8 ± 3.2	0.6 (− 1.1, 2.1)
Anthropometry						
Height (cm)	180.4 ± 7.3	180.2 ± 7.8	0.2 (− 3.7, 4.2)	167.8 ± 5.2	166.4 ± 5.5	1.5 (− 1.4, 4.3)
Weight (kg)	77.0 ± 16.0	74.3 ± 11.7	3.0 (− 4.8, 10.7)	63.8 ± 10.0	61.2 ± 11.4	2.7 (− 3.1, 8.4)
BMI (kg/m^2^)	23.7 ± 4.8	22.9 ± 3.6	0.8 (− 1.6, 3.2)	22.6 ± 3.3	22.0 ± 3.3	0.6 (− 1.2, 2.4)
Bone mineral content (BMC; g)						
Total body less head	2657.8 ± 518.6	2606.7 ± 376.2	63.5 (− 181.3, 308.2)	2064.3 ± 342.4	1896.3 ± 264.7	168.7 (− 2.9, 340.2)
Arms	439.9 ± 74.5	436.5 ± 61.0	4.8 (− 31.9, 41.5)	304.1 ± 41.2	297.8 ± 38.8	6.4 (− 15.6, 28.3)
Legs	1214.1 ± 224.5	1206.4 ± 167.4	13.7 (− 91.9, 119.3)	944.5 ± 155.2	885.0 ± 119.7	59.9 (− 17.5, 137.4)
Spine	257.1 ± 65.4	244.6 ± 58.3	14.0 (− 18.4, 46.4)	221.1 ± 48.5	194.6 ± 36.3	26.6 (2.5, 50.7)
Hip – femoral neck	6.4 ± 1.2	6.2 ± 0.8	0.2 (− 0.4, 0.8)	5.2 ± 0.8	4.8 ± 0.7	0.3 (− 0.1, 0.8)
Bone mineral density (BMD; g/cm^2^)						
Total body less head	1.13 ± 0.12	1.21 ± 0.10	0.01 (− 0.05, 0.07)	1.04 ± 0.10	1.01 ± 0.07	0.03 (− 0.02, 0.08)
Arms	0.92 ± 0.11	0.88 ± 0.11	0.03 (− 0.02, 0.09)	0.79 ± 0.09	0.77 ± 0.08	0.02 (− 0.02, 0.07)
Legs	1.39 ± 0.15	1.38 ± 0.13	0.02 (− 0.06, 0.09)	1.25 ± 0.13	1.22 ± 0.08	0.03 (− 0.03, 0.09)
Spine	1.10 ± 0.14	1.09 ± 0.10	0.01 (− 0.05, 0.08)	1.06 ± 0.13	1.02 ± 0.12	0.05 (− 0.02, 0.11)
Hip – femoral neck	1.16 ± 0.18	1.15 ± 0.15	0.01 (− 0.08, 0.10)	1.10 ± 0.15	1.04 ± 0.13	0.06 (− 0.01, 0.14)
Muscle strength – Peak torque (Nm)						
Knee flexion 60°	112.6 ± 23.8	113.5 ± 24.2	− 0.5 (− 13.1, 12.1)	71.5 ± 16.1	68.4 ± 12.2	3.1 (− 5.0, 11.2)
Knee flexion 180°	82.9 ± 21.2	82.5 ± 18.6	0.7 (− 10.0, 11.3)	51.4 ± 11.3	49.1 ± 11.5	2.3 (− 3.9, 8.5)
Muscle strength – Peak torque TBW (Nm/kg)*100						
Knee flexion 60°	148.5 ± 23.4	150.6 ± 27.7	− 2.0 (− 15.4, 11.3)	110.9 ± 27.2	113.9 ± 21.5	− 3.1 (− 16.9, 10.6)

Data are presented as numbers (n), proportions (%), means ± standard deviations, or mean age-adjusted differences with 95% confidence intervals

## Discussion

In this small cohort study, in which we followed children 4 years after termination of an intervention with daily school PA, we found indications that the muscle strength benefits (but not bone mass benefits) were eroded with retirement, but also indications that accrual of both bone mass and muscle strength during the entire 11-year study period nevertheless was higher in the intervention group than in the control group.

In previous POP reports we have found that the intervention program with moderately intense daily school PA during the first nine school years (including the pubertal period) in both sexes is associated with greater gain in bone mass and a better musculoskeletal composite score for fracture, compared to children with school PA only 1–2 times per week [6]. The benefits seem clinically relevant, as we found an inverse correlation between the number of years with daily school PA and annual fracture incident rate ration (IRR) [7, 8]. Previous POP reports also indicate the importance of following the children during and through the pubertal period. For example, when 40% of the children were in puberty (below Tanner stage V), we were able to identify benefits only for girls [7]. In contrast, when all children had passed puberty (were in Tanner stage V), we found benefits in both sexes [6].

Before making recommendations on PA intervention in childhood as a population strategy to counteract low bone mass and inferior muscle strength in adulthood, we must be able to show residual musculoskeletal benefits after the termination of the program. Some studies infer that reduced level of PA is followed by greater loss in bone mass than expected with age [16, 21, 22]. Others oppose this view, with reports indicating that exercise-induced bone mass benefits in young years are retained in adulthood [12–16, 23, 24], and that children with a high level of PA during growth have a lower fracture incidence in adulthood [12, 13, 15, 16]. These studies, however, included individuals with a self-selected high level of PA, who already at baseline had high bone mass and superior muscle function [12–16, 21–24]. We therefore cannot rule out that selection bias was present and contributed to the results. Healthy, strong and physically fit individuals may choose PA as a spare-time activity to a greater extent since they are probably good at sport. The higher neuromuscular function and higher bone mass in this group may be due to genetic factors and not a causal relationship between high PA and superior musculoskeletal traits. This is in contrast to our study, were at the baseline examination we found a similar level of PA and muskuloskeletal traits without indications of selection bias.

Yet we are still not able to draw causal inferences regarding PA intervention during childhood and adolescence and beneficial musculoskeletal traits in young adulthood, since the former intervention group continued to choose to have a higher level of PA after termination of the intervention [26]. It thus seems as if a 9-year intervention with daily school PA may result in a more physically active lifestyle [26] and the causal link between intervention and adult benefits may at least partly be between the more physical active lifestyle with higher duration of PA after termination of the intervention rather than the PA intervention in school itself.

Another possibility is that there may be a causal relationship between factors associated with the intervention program as well as the beneficial gain. For example, children (and the parents) in the intervention group may have gained a greater knowledge and interest in health-related matters during the initiation of the intervention, and due to this voluntarily changed other habits, such as nutritional intake or method of transportation. It could thus hypothetically be an improved nutritional intake or that the children started to walk or cycle to school instead of taking the bus, or started using stairs instead of elevators, that provides the causal link to the improved musculoskeletal gains. In the clinical perspective the causal link is of less importance, however, as we nevertheless did reach the goals of the intervention program.

Study strengths include the prospective, controlled and population-based study design and the fact that all participants were followed from Tanner stage I to V. Study limitations include the small sample size and high drop-out frequency, resulting in risks of both selection bias and type II errors. However, the drop-out analyses did not indicate any substantial selection bias, and to minimize the risk of type II error we avoided subgroup analyses (i.e., sex-specific evaluations). This is also the reason why we chose to present only sex-specific data at age 19 without inferential statistics. Even if the gains during the entire study period in absolute values are higher in the intervention group in most measurements, the only statistically significant differences are for bone mass gain in arms and spine, and for muscle strength in PT_flex180_TBW. We thus need to be extra careful with inferences, and this is the reason why we only state that we found indications of beneficial effects. It would also have been advantageous to include a longer follow-up period, as the musculoskeletal benefits may be lost in a longer perspective. Children in this study were predominantly of Caucasian ethnicity, living in a socioeconomic middle-class area. Transferring the inferences to children with other ethnic backgrounds or living in other socioeconomic areas may thus be questionable. The lack of individual randomization is another weakness that we were unable to address due to practical problems with the school schedule and difficulties keeping a strict randomization over 9 years. Further limitations include self-evaluation of the duration of organized leisure-time PA, without registration of type of activity, duration and/or level of PA during play. It would also have been advantageous to have objectively registered PA measured by accelerometers. The use of surrogate end points in this study rather than fractures should also be regarded as a weakness.

In summary, we found that beneficial bone mass accrual and muscle strength gain remained into young adulthood 4 years after the termination of a daily school-based PA intervention program that had been ongoing all 9 compulsory school years. Due to the study limitations, inferences should serve only as indications that daily school PA may counteract low bone mass and inferior muscle strength later in life. Results need to be verified in larger studies with a longer follow-up.

## Data Availability

Data can be provided by the corresponding author at time of publication.

## Code Availability

Statistical analyses were done by Statistica® version 12.0 (Statsoft Inc®).

## References

[1] Cooper C, Dennison EM, Leufkens HG, Bishop N, van Staa TP (2004). Epidemiology of childhood fractures in Britain: a study using the general practice research database. J Bone Miner Res.

[2] Johnell O, Kanis J (2005). Epidemiology of osteoporotic fractures. Osteoporos Int.

[3] Borgstrom F, Zethraeus N, Johnell O, Lidgren L, Ponzer S, Svensson O, Abdon P, Ornstein E, Lunsjo K, Thorngren KG (2006). Costs and quality of life associated with osteoporosis-related fractures in Sweden. Osteoporos Int.

[4] Clark EM, Ness AR, Bishop NJ, Tobias JH (2006). Association between bone mass and fractures in children: a prospective cohort study. J Bone Miner Res.

[5] Karlsson MK, Vonschewelov T, Karlsson C, Coster M, Rosengen BE (2013). Prevention of falls in the elderly: a review. Scand J Public Health.

[6] Cronholm F, Lindgren E, Rosengren BE, Dencker M, Karlsson C, Karlsson MK (2020). Daily school physical activity from before to after puberty improves bone mass and a musculoskeletal composite risk score for fracture. Sports.

[7] Coster ME, Rosengren BE, Karlsson C, Dencker M, Karlsson MK (2016). Effects of an 8-year childhood physical activity intervention on musculoskeletal gains and fracture risk. Bone.

[8] Fritz J, Coster ME, Nilsson JA, Rosengren BE, Dencker M, Karlsson MK (2016). The associations of physical activity with fracture risk–a 7-year prospective controlled intervention study in 3534 children. Osteoporos Int.

[9] Coster ME, Fritz J, Nilsson JA, Karlsson C, Rosengren BE, Dencker M, Karlsson MK (2017). How does a physical activity programme in elementary school affect fracture risk? A prospective controlled intervention study in Malmo. Sweden BMJ Open.

[10] Linden C, Ahlborg HG, Besjakov J, Gardsell P, Karlsson MK (2006). A school curriculum-based exercise program increases bone mineral accrual and bone size in prepubertal girls: two-year data from the pediatric osteoporosis prevention (POP) study. J Bone Miner Res.

[11] Behringer M, Gruetzner S, McCourt M, Mester J (2014). Effects of weight-bearing activities on bone mineral content and density in children and adolescents: a meta-analysis. J Bone Miner Res.

[12] Karlsson MK, Linden C, Karlsson C, Johnell O, Obrant K, Seeman E (2000). Exercise during growth and bone mineral density and fractures in old age. Lancet.

[13] Tveit M, Ahlborg H, Rosengren B, Nilsson J-Å, Karlsson M (2010). Bone loss and fracture risk after high level of physical activity at growth and young adulthood. J Bone Miner Res.

[14] Tveit M, Rosengren BE, Nilsson JA, Ahlborg HG, Karlsson MK (2013). Bone mass following physical activity in young years: a mean 39-year prospective controlled study in men. Osteoporos Int.

[15] Tveit M, Rosengren BE, Nilsson JA, Karlsson MK (2015). Exercise in youth: high bone mass, large bone size, and low fracture risk in old age. Scand J Med Sci Sports.

[16] Nordstrom A, Karlsson C, Nyquist F, Olsson T, Nordstrom P, Karlsson M (2005). Bone loss and fracture risk after reduced physical activity. J Bone Miner Res.

[17] Kannus P, Haapasalo H, Sankelo M, Sievanen H, Pasanen M, Heinonen A, Oja P, Vuori I (1995). Effect of starting age of physical activity on bone mass in the dominant arm of tennis and squash players. Ann Intern Med.

[18] Bailey DA, McKay HA, Mirwald RL, Crocker PR, Faulkner RA (1999). A six-year longitudinal study of the relationship of physical activity to bone mineral accrual in growing children: the university of Saskatchewan bone mineral accrual study. J Bone Miner Res.

[19] Hui SL, Slemenda CW, Johnston CC (1990). The contribution of bone loss to postmenopausal osteoporosis. Osteoporos Int.

[20] Hernandez CJ, Beaupre GS, Carter DR (2003). A theoretical analysis of the relative influences of peak BMD, age-related bone loss and menopause on the development of osteoporosis. Osteoporos Int.

[21] Gustavsson A, Olsson T, Nordstrom P (2003). Rapid loss of bone mineral density of the femoral neck after cessation of ice hockey training: a 6-year longitudinal study in males. J Bone Miner Res.

[22] Valdimarsson O, Alborg HG, Duppe H, Nyquist F, Karlsson M (2005). Reduced training is associated with increased loss of BMD. J Bone Miner Res.

[23] Ravi S, Kujala UM, Tammelin TH, Hirvensalo M, Kovanen V, Valtonen M, Waller B, Aukee P, Sipila S, Laakkonen EK (2020). Adolescent sport participation and age at menarche in relation to midlife body composition, bone mineral density, fitness, and physical activity. J Clin Med.

[24] Bass S, Pearce G, Bradney M, Hendrich E, Delmas PD, Harding A, Seeman E (1998). Exercise before puberty may confer residual benefits in bone density in adulthood: studies in active prepubertal and retired female gymnasts. J Bone Miner Res.

[25] Tanner JM, Whitehouse RH (1976). Clinical longitudinal standards for height, weight, height velocity, weight velocity, and stages of puberty. Arch Dis Child.

[26] Lahti A, Rosengren BE, Nilsson JA, Karlsson C, Karlsson MK (2018). Long-term effects of daily physical education throughout compulsory school on duration of physical activity in young adulthood: an 11-year prospective controlled study. BMJ Open Sport Exerc Med.

